# Comparative genomic analysis of an emerging Pseudomonadaceae member, *Thiopseudomonas alkaliphila*

**DOI:** 10.1128/spectrum.04157-23

**Published:** 2024-06-27

**Authors:** Zachary M. Burcham

**Affiliations:** 1 Department of Microbiology, University of Tennessee, Knoxville, Tennessee, USA; Indian Institute of Technology Hyderabad, Hyderabad, Telangana, India

**Keywords:** comparative genomics, environmental microbiology, gut microbiota, decomposition, emerging

## Abstract

*Thiopseudomonas alkaliphila*, an organism recently classified within the Pseudomonadaceae family, has been detected in diverse sources such as human tissues, animal guts, industrial fermenters, and decomposition environments, suggesting a diverse ecological role. However, a large knowledge gap exists in how *T. alkaliphila* functions. In this comparative genomic analysis, adaptations indicative of habitat specificity among strains and genomic similarity to known opportunistic pathogens are revealed. Genomic investigation reveals a core metabolic utilization of multiple oxidative and non-oxidative catabolic pathways, suggesting adaptability to varied environments and carbon sources. The genomic repertoire of *T. alkaliphila* includes secondary metabolites, such as antimicrobials and siderophores, indicative of its involvement in microbial competition and resource acquisition. Additionally, the presence of transposases, prophages, plasmids, and Clustered Regularly Interspaced Short Palindromic Repeats-Cas systems in *T. alkaliphila* genomes suggests mechanisms for horizontal gene transfer and defense against viral predation. This comprehensive genomic analysis expands our understanding on the ecological functions, community interactions, and potential virulence of *T. alkaliphila*, while emphasizing its adaptability and diverse capabilities across environmental and host-associated ecosystems.

**IMPORTANCE:**

As the microbial world continues to be explored, new organisms will emerge with beneficial and/or pathogenetic impact. *Thiopseudomonas alkaliphila* is a species originally isolated from clinical human tissue and fluid samples but has not been attributed to disease. Since its classification, *T. alkaliphila* has been found in animal guts, animal waste, decomposing remains, and biogas fermentation reactors. This is the first study to provide an in-depth view of the metabolic potential of publicly available genomes belonging to this species through a comparative genomics and draft pangenome calculation approach. It was found that *T. alkaliphila* is metabolically versatile and likely adapts to diverse energy sources and environments, which may make it useful for bioremediation and in industrial settings. A range of virulence factors and antibiotic resistances were also detected, suggesting *T. alkaliphila* may operate as an undescribed opportunistic pathogen.

## INTRODUCTION

*Thiopseudomonas alkaliphila* (NCBI:txid 1697053), previously *Oblitimonas alkaliphila*, was originally isolated from the bodily fluids and tissues of eight human patients from 1969 to 1979; however, *T. alkaliphila* is not known to cause disease in healthy individuals. These isolates remained unidentified until recent diagnostics and biochemical testing placed these strains within the Pseudomonadaceae family under the novel genus and species *Thiopseudomonas alkaliphila* (1, 2). *T. alkaliphila* are Gram-negative, aerobic/microaerophilic, non-motile, alkali-tolerant, and halo-tolerant, and exhibit both oxidase and catalase activities. Due to the relatively recent classification of *T. alkaliphila,* there is still a large gap in our knowledge of this organism; therefore, there is a need to gain a deeper understanding of its role in ecosystem function and industrial use potential. As of July 2023, only 12 genomes were publicly available in the NCBI Assembly database; however, two genomes were excluded from the NCBI RefSeq collection due to missing tRNA genes (genome assembly ASM126723v1), or the genome was too small and fragmented (genome assembly ASM236031v1). Based on the previous annotation performed by the NCBI Prokaryotic Genome Annotation Pipeline (PGAP) (3), average genome size was 2,402,779 bp and on average contained 2,316.3 genes which included 2,211.2 protein-coding and 65.6 non-coding genes. Average genome completeness was 83.73% and average genome contamination was 1.52%. Genome metadata and assembly statistics can be found in Table 1 and Table S1. The comparative genome and pangenome analyses in this study were only performed on the 10 high-quality genomes (*N* = 10) generated from isolates included in the NCBI RefSeq collection. Due to the small number of available genomes, the pangenome analysis presented here should only be considered a draft pangenome but will serve as a resource as more strains are identified. The strains were isolated from eight human patients from urine, leg tissue, foot wound, lung tissue, and deep liver tissue (*n* = 7). The remaining isolates (*n* = 3) were obtained from chicken intestines within China in 2019 due to tetracycline-resistance selection and the presence of the *tetX* tetracycline-resistance gene (4).

**TABLE 1:** Overview of *Thiopseudomonas alkaliphila* genome information derived from NCBI RefSeq^*a*^

Strain	Genome size (bp)	No. scaffolds	N50 (Mb)	% GC	No. genes	No. coding	No.non-coding	Completeness/ contamination	Source	Host
B4199^T^	2,494,031	1	2.5	47	2422	2320	73	84.58/0.9	Deep liver	Human
C6819	2,272,143	1	2.3	47	2199	2100	64	81.22/2.96	Leg wound	Human
C6918	2,312,033	1	2.3	47.5	2222	2124	74	83.99/1.73	Lung tissue	Human
D3318^R^	2,294,397	1	2.3	47	2202	2109	69	84.48/0.72	Foot wound	Human
E1086	2,391,994	1	2.4	47	2281	2174	69	82.72/3.02	Leg	Human
E1148	2,377,259	1	2.4	47	2290	2177	73	82.37/2.43	Urine	Human
E5571	2,397,029	1	2.4	47	2317	2203	73	84.41/0.59	Urine	Human
DF95-5	2,475,608	60	0.17	48	2370	2266	53	84.72/0.59	Intestine	Chicken
DF46-2-2	2,572,910	40	0.35	47.5	2473	2368	54	84.3/0.92	Intestine	Chicken
DF92-3	2,440,383	84	0.3	47	2387	2271	54	84.52/1.32	Intestine	Chicken

^*a*^ Full table can be found as Table S1. ^T^ and ^R^ denote type strain and NCBI reference genome, respectively.

In recent years, *T. alkaliphila* has been increasingly identified in terrestrial and agricultural systems. Operational taxonomic units (OTUs) with 96% similarity to *T. alkaliphila* have been associated with the anaerobic digestion of wheat husks where it is hypothesized to catabolize glucose and xylose (5). It is suspected that this OTU similar to *T. alkaliphila* may have a role in ammonolysis leading to the breakage of ester bonds connecting hemicellulose and lignin, a process beneficial to methane production (5, 6). *T. alkaliphila* has also been found as a member of the animal gut microbiome in animals such as giant panda cubs (7) and chickens (4). *T. alkaliphila* is further associated with animal waste including excrement from manure-borne black soldier fly larvae (8), farm animal manure (9, 10), and decomposing animal/human remains (11–13). These studies suggest that *T. alkaliphila* may play an important role in the recycling of nutrients derived from organic matter through various ecosystems, particularly during the process of decomposition. *T. alkaliphila* has been identified as a potential key member in a conserved interdomain microbial network responsible for the decomposition of cadavers across distinct environments (13). A metagenome-assembled genome assigned to *T. alkaliphila* was predicted to be responsible for amino acid metabolism and metabolite cross-feeding during decomposition. These processes are critical to fueling ecosystem functions, such as plant primary productivity, and sustaining the global food web. Furthermore, *T. alkaliphila* has been detected as a prominent microbe producing acetic and lactic acids by glucose fermentation in trickling filter bed reactors that convert organic matter to biogas (5, 14), suggesting a potential industrial role for *T. alkaliphila* to generate alternative, renewable energy sources. In this study, a comparative genome analysis to evaluate the genomic content and genomic potential of *T. alkaliphila* strains was performed and used to establish the current state of the *T. alkaliphila* pangenome to serve as a jumping point to gain better understanding of this potential ecologically and industrially beneficial organism.

## RESULTS AND DISCUSSION

### Genomic similarity and phylogenetic placement

**Fig 1:**
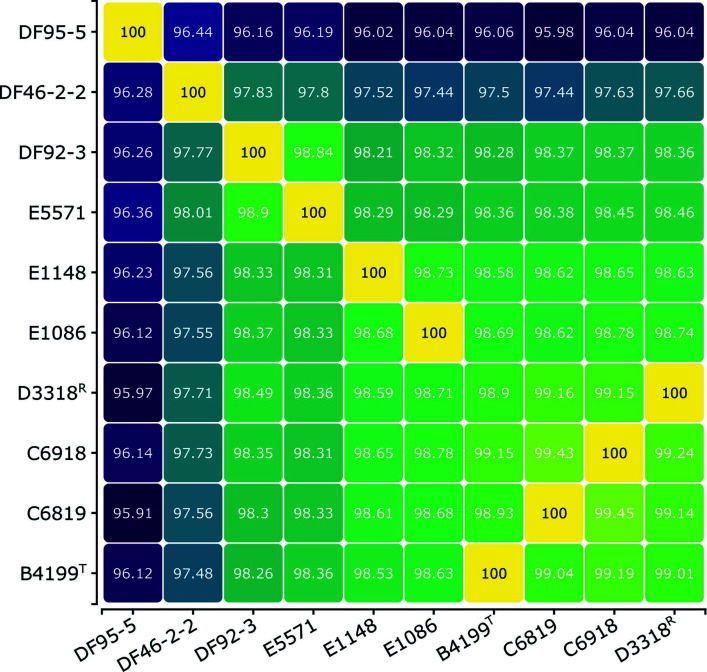
Genomic content comparison shows relatedness of study *T. alkaliphila* strains via pairwise ANI calculations by FastANI. Strains are clustered by ANI. ^T^ and ^R^ denote type strain and NCBI reference genome, respectively.

Genome metadata and assembly statistics can be found in Table 1 and Table S1. A 95% average nucleotide identity (ANI) threshold is commonly used to demarcate species from each other (15). Here, the pairwise ANI for analyzed strains ranged from 95.91% to 100%, placing all genomes within this study under the species *T. alkaliphila* (Fig. 1; Table S2). *T. alkaliphila* strains isolated from humans clustered separately from chicken isolates. Chicken isolate DF95-5 and human isolate C6819 were the most dissimilar strains in pairwise comparison. This suggests that genomic differences between strains may be partially explained by habitat/host.

To explore the relatedness of *T. alkaliphila* strains in the context of closely related type strains, two phylogenetic trees were constructed using the Type Strain Genome Server (TYGS; see Materials and Methods). In brief, for the first tree, all *T. alkaliphila* whole genomes were compared against all 20,806 reference type strain genomes available in the TYGS database. Type strains are the first strains described when a new strain is introduced. Comparisons with established type strains are often mandatory when classifying novel strains as they form the backbone of prokaryotic species and subspecies systematics (16). For the second tree, an additional set of 10 closely related type strains was determined via the 16S rRNA gene sequences of each of TYGS database type strains. The 16S rRNA is commonly used for bacterial and archaeal taxonomic classification and phylogenetic placement due to its highly conserved nature across prokaryotes. For both the whole-genome and 16S rRNA tree, the best 50 matching type strains for each *T. alkaliphila* genome was used to calculate distances using the Genome BLAST Distance Phylogeny (GBDP) approach. These distances were used to determine the 10 closest type strain genomes for each of the *T. alkaliphila* strains and place them in the phylogenetic tree.

**Fig 2:**
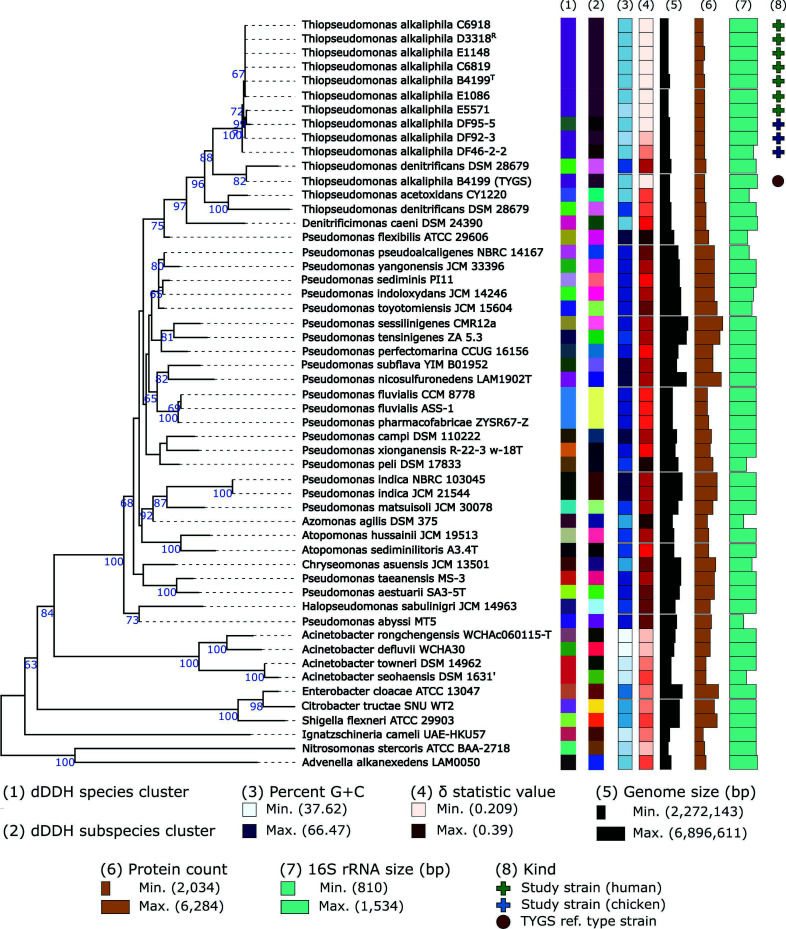
Phylogenetic tree inferred from GBDP distances calculated from 16S rRNA gene sequences demonstrates closest related type strains to *T. alkaliphila* strains along with information on genomic content. The branch lengths are scaled in terms of GBDP distance formula *d_5_*. The numbers above branches are GBDP pseudo-bootstrap support values >60% from 100 replications, with an average branch support of 66.5%. The tree was rooted at the midpoint. Leaf labels are annotated by affiliation to species (1) and subspecies (2) clusters, percent genomic G + C content (3), δ statistic value (4), genome sequence size (5), protein count (6), 16S rRNA gene size (7), and the kind of strain (8). Genomes with high δ values correspond to sequence incompleteness/contamination and highlights specific genome sequences that may impact the reliability of the tree. ^T^ and ^R^ denote type strain and NCBI reference genome, respectively.

The 16S rRNA inferred tree clustered all *T. alkaliphila* strains in this study into the same clade (Fig. 2). In support of the pairwise ANI results, this clade was further divided into subclades which primarily split the strains based on host. The exception to this was strain E5571 which clustered in a subclade with DF95-5. Type-based species and subspecies clustering using a 70% and 79% digital DNA-DNA hybridization (dDDH) values, respectively, assigned all *T. alkaliphila* strains under the same species and mostly the same subspecies. Strain DF95-5 was the only strain considered as a different species cluster. This result is unsurprising as DF95-5 has been the strain with consistently the least relatedness to the other strains and is just within the 95% ANI threshold for species placement (Fig. 1). The type-strain species most closely related to *T. alkaliphila* based on 16S rRNA are *Thiopseudomonas denitrificans* DSM 28679, *Thiopseudomonas acetoxidans* CY1220, and *Denitrificimonas caeni* (synonym for *Pseudomonas caeni*) DSM 24390 (Fig. 2). *T. acetoxidans* is a novel species recently isolated from the anaerobic fermentation of food waste at a treatment plant (17). *T. denitrificans* and *D. caeni* are both denitrifying bacteria isolated from anerobic, activated-sludge bioreactors (18–20). All three strains are classified as facultative anaerobes which is defined as the ability to grow via aerobic respiration when oxygen is available or fermentation in anaerobic conditions. However, despite the genetic similarity and possible detection of *T. alkaliphila* in anerobic bioreactor, *T. alkaliphila* has not been shown to grow under anaerobic conditions but could grow microaerophilicly (2, 5, 6). It has been proposed that the species *D. caeni, T. alkaliphila*, and *T. denitrificans* compose a monophyletic group and should be integrated into the same genus, *Thiopseudomonas* (19), which would now likely include *T. acetoxidans*.

**Fig 3:**
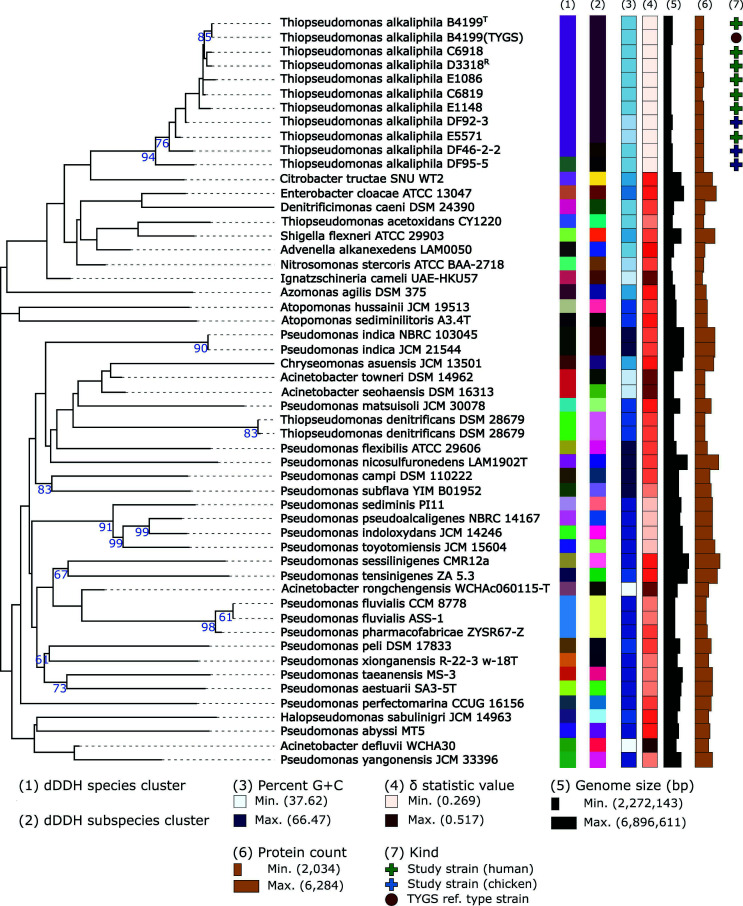
Phylogenetic tree inferred from GBDP distances calculated from the whole-genome sequence demonstrates closest related type strains to *T. alkaliphila* strains along with information on genomic content. The branch lengths are scaled in terms of GBDP distance formula *d_5_*. The numbers above branches are GBDP pseudo-bootstrap support values >60% from 100 replications, with an average branch support of 34.3%. The tree was rooted at the midpoint. Leaf labels are annotated by affiliation to species (1) and subspecies (2) clusters, percent genomic G + C content (3), δ statistic value (4), genome sequence size (5), protein count (6), and the kind of strain (7). Genomes with high δ values correspond to sequence incompleteness/contamination and highlights specific genome sequences that may impact the reliability of the tree. ^T^ and ^R^ denote type strain and NCBI reference genome, respectively.

An expansion of the phylogenetic comparison to incorporate the whole genome revealed similar clustering for the *T. alkaliphila* strains, including the host differentiation, apart from strain E5571 and unique species cluster for strain DF95-5 (Fig. 3). Inter-species genomic comparison underscores that *T. alkaliphila* genomes are relatively small and have lower G + C content percentage and protein counts than closely related type strains. Reduced genomes are commonly associated with commensalism-based reductive evolution (i.e., Black Queen hypothesis) that theorizes that natural selection can lead to the loss of energetically costly genes and functions that produce resources if these resources are provided by the environment, host, or other microbial community members (21, 22). The reduced genome of *T. alkaliphila* may also be indicative of involvement in complex interaction networks such as the cross-feeding potential of *T. alkaliphila* detected within a microbial decomposer network (13). *T. acetoxidans* and *D. caeni* were both still considered to have a high relatedness to *T. alkaliphila*; however, *T. denitrificans* was placed in a separate clade which shows distant divergence. Interestingly, two new type strains were placed near *T. alkaliphila* when the whole genome is considered, *Citrobacter tructae* SNU WT2 and *Enterobacter cloacae* ATCC 13047. *C. tructae* is a novel *Citrobacter* species isolated from the kidney of a diseased rainbow trout (23). *C. tructae* demonstrated antibiotic resistance to β-lactams, quinolone, and aminoglycosides in part due to plasmid-encoded resistance genes. *Citrobacter* are not typically associated with diseases; however, some species such as *Citrobacter freundii* have been associated with opportunistic infections in immunocompromised populations. *Citrobacter* species inhabit diverse niches such as the animal/human gut and soil and water sources. *E. cloacae* ATCC 13047 was isolated from human cerebrospinal fluid (24). *E. cloacae* strains are common in nature, but are primarily known for being human opportunistic pathogens, largely associated with hospital-acquired, nosocomial infections (25). Currently, *E. cloacae* strains are a grave concern to care-giving industries due to its heavy-metal and multidrug resistances, including recent emergences of resistance to carbapenems, which are typically used as a “last-ditch effort” to treat infections.

The observed discrepancy in clustering patterns between the 16S rRNA and whole-genome inferred trees could be due to differences in evolutionary rates, horizontal gene transfer events, incomplete lineage sorting, and gene duplication/loss. This indicates that while using phylogenetic markers such as 16S rRNA may highlight ancestral similarity and conserved function, whole-genome alignments incorporate functional diversity from the shell and cloud functional capacities outside of core function. In fact, the ability to predict function based on 16S rRNA gene sequences is a contested topic in microbiome research due, in part, to the discrepancies shown here. The taxa with similar 16S rRNA sequences to *T. alkaliphila* are primarily associated with decomposition process of animal and plant products, which is line with environments *T. alkaliphila* has been detected. However, a whole-genome comparison reveals that *T. alkaliphila* is also genetically similar to both known opportunistic pathogens (e.g., *Citrobacter* and *Enterobacter*). *T. alkaliphila* is currently considered a biosafety level 1 (BSL-1) organism, meaning it is not known to cause disease in healthy populations. Opportunistic pathogens are traditionally associated with non-healthy populations. At the time of this study, there are no published data measuring the pathogenicity of *T. alkaliphila* strains. However, it is important to note that the human-derived strains in this study were clinical isolates from human tissues and wounds. While the isolation of *T. alkaliphila* does not necessarily correspond to causation of disease, the presence of *T. alkaliphila* in wounds may indicate a role, even if nonpathogenic, in disease ecology not shown by other *Thiopseudomonas* species.

### Central metabolism annotation

**Fig 4:**
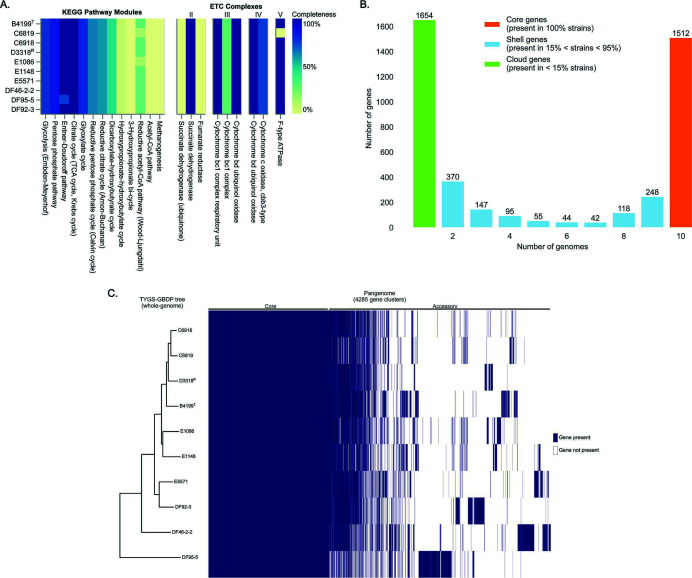
Metabolic annotation and pangenome calculation reveal core and accessory metabolisms of *T. alkaliphila* strains. (**A**) Completeness of KEGG metabolic pathway modules for carbon utilization, energy acquisition, and the electron transport chain (ETC) complexes across strains. (**B**) The number of shared genes across genomes and classification of core, shell, or cloud genes based on percent presence. (**C**) Heatmap of the core and accessory genomes sorted by the TYGS-GBDP whole-genome phylogenetic tree shows strain-specific variation of accessory genome. ^T^ and ^R^ denote type strain and NCBI reference genome, respectively.

Profiling of the coverage of Kyoto Encyclopedia of Genes and Genomes (KEGG) pathway modules reveals *T. alkaliphila* are similar in how they utilize carbon and generate energy (Fig. 4A; Tables S3 and S4). Biochemical testing on the human isolates shows that *T. alkaliphila* can oxidize glucose (2). A genomic investigation of *T. alkaliphila* capacity to catabolize glucose via glycolysis showed the presence of an incomplete pathway (7/9 KEGG pathway module steps). The genes for the cleavage of fructose-1,6-bisphosphate and the core module involving catabolism of three-carbon compounds is compete, suggesting *T. alkaliphila* can use the glycolysis pathway to obtain energy from intermediates of the cycle. However, *T. alkaliphila* genomes are absent of hexokinase/glucokinase and phosphofructokinase genes which are responsible for ATP phosphorylation of glucose to glucose 6-phosphate and phosphorylation of fructose 6-phosphate to fructose 1,6-bisphosphate. These are the two primary energy usage steps in the beginning of glycolysis when traditional ATP-dependent transport and intracellular phosphorylation occur. Some species that are missing hexokinase and glucokinase commonly use a phosphotransferase system (PTS) that couples the transport and phosphorylation of carbohydrates, bypassing the need for hexokinase and glucokinase to complete glycolysis (26), but *T. alkaliphila* does not contain sugar PTS so it seems unlikely this mechanism is utilized. Furthermore, *T. alkaliphila* does not have known alternate enzymes that use inorganic polyphosphate for the phosphorylation of glucose or ability to convert glucose to gluconate which can enter the Entner-Doudoroff (ED) pathway. Therefore, it is not clear how *T. alkaliphila* initially processes glucose to generate pathway intermediates. One possible avenue of investigation includes the use of alternate hexokinase/glucokinase-like enzymes not currently characterized.

Given the presence of phosphorylated glucose (glucose 6-phosphate), either generated by uncharacterized mechanisms or acquired from the environment, *T. alkaliphila* can bypass the need for phosphofructokinase by shuttling glucose 6-phosphate to the ED pathway (4/4 module steps in all strains except DF95-5) (Fig. 4A; Tables S3 and S4). The ED pathway is commonly found in bacteria, particularly Gram-negative facultative anaerobes. It is presumed to have evolved earlier than glycolysis due to the simplified pathway and production of fewer ATP molecules. Obligate anaerobes predominantly use glycolysis over the ED pathway due to the increased ATP yield from glycolysis and inability to generate ATP via oxidative phosphorylation in the electron transport chain (ETC) (27). Aerobes and facultative aerobes can take advantage of the reduced protein cost of the ED pathway by acquiring large amounts of ATP from pyruvate produced for the citric acid or tricarboxylic acid (TCA) cycle and subsequent oxidative phosphorylation. A complete TCA cycle and components of the ETC are found in all *T. alkaliphila* strains, suggesting *T. alkaliphila* relies on oxidative phosphorylation when oxygen is present. The *T. alkaliphila* ETC does not contain the enzymes of complex I (e.g., NADH dehydrogenase), but rather contains succinate dehydrogenase (complex II). Succinate dehydrogenase catalyzes the conversion of succinate to fumarate during the citrate cycle while transferring electrons to the electron carrier, ubiquinone, to form ubiquinol. The cytochrome bc1 complex and cytochrome bd ubiquinol oxidase (complex III) transfer the electrons from ubiquinol to a cytochrome (e.g., heme) and use oxygen as a final electron acceptor to generate water and protons through oxidative phosphorylation (complex IV). Finally, the protons flow through the F-type ATPase complex (complex V) to generate ATP. *T. alkaliphila* strain C6819 was the only strain that did not contain genes for any type of ATPase. The lack of an ATP synthase complex is highly unlikely in bacteria that are not obligate intracellular pathogens; therefore, it is more likely that this portion of the genome was absent from the assembly considering strain C6819 had the lowest number of coding genes and lowest genome completeness in the data set (Table 1). *T. alkaliphila* can also perform fermentation by pyruvate generation through glycolysis followed by lactate production derived from pyruvate by lactate dehydrogenase while ethanol can be produced with alcohol dehydrogenase.

Other energy-yielding pathways detected include most of the pentose phosphate pathway (6/7 module steps) for oxidizing glucose (Fig. 4A; Tables S3 and S4); however, this pathway is primarily involved in the biosynthesis of 5-carbon sugar phosphates and a precursor to generate nucleotides rather than relied on for energy acquisition. The glyoxylate cycle is an anabolic pathway analogous to the TCA cycle which converts 2-carbon resources, such as acetate and acetyl-coenzyme A (CoA), to malate then oxaloacetate. Oxaloacetate can be used as an intermediate in the TCA cycle or for the biosynthesis of glucose and other carbohydrates when simple sugars are not readily available. Other than being converted to oxaloacetate, malate can be converted to pyruvate. The majority of the glyoxylate cycle is present in all strains (4/5 module steps); the missing component is the traditional glyoxylate cycle malate dehydrogenase (MDH1 or MDH2) for oxaloacetate generation. However, *T. alkaliphila* contains the unique enzyme malate:quinone oxidoreductase (MQO) that can convert malate to oxaloacetate. MQO is part of both the electron transfer chain and the citric acid cycle, similar to succinate dehydrogenase (28). A component of the acetate assimilation pathway is present in all strains granting the ability to convert free acetate to acetyl-phosphate via acetate kinase and subsequently transfer the acetyl group to CoA via acetyl transferase to form acetyl-CoA for the glyoxylate and TCA cycles. This strengthens the hypothesis that *T. alkaliphila* can likely use acetate as a readily available carbon source. The presence of an acetate assimilation pathway and the glyoxylate cycle would give *T. alkaliphila* greater metabolic versatility in nutrient use for energy and growth.

The ability to oxidize thiosulfate to sulfate by the sulfur oxidizing (SOX) complex was detected in all strains (Fig. 4A; Tables S3 and S4). Thiosulfate oxidation is a process that allows thiosulfate to serve as an alternate electron donor for bacterial respiration. The SOX complex is commonly associated with environmental organisms in sulfur-rich environments. For example, sulfur-rich compounds are prevalent during organic-matter decomposition (29); therefore, *T. alkaliphila* may gain energy from and have a role in facilitating the mineralization of sulfur within ecosystems. This process also prevents the accumulation of toxic sulfur compounds, like hydrogen sulfide, which can be harmful to many forms of life if present in high concentrations. Sulfur oxidation is not a typical feature of obligate gut bacteria. One exception is *Salmonella enterica* serotype Typhimurium which has been shown to use tetrathionate as an electron acceptor during gut inflammation (30). Interestingly, half of the strains, all of which were human isolates, are predicted to convert tetrathionate to thiosulfate. A repertoire of sulfur oxidation suggests beneficial mechanisms for *T. alkaliphila* to grow in sulfur-rich environments, the gut during inflammation, and may serve to aid transition between living/dead host-associated and terrestrial ecosystems. Another enzyme of ecological importance is arsenate reductase glutaredoxin which was present in multiple copies in all *T. alkaliphila* strains. Arsenate is a form of the toxic metalloid arsenic that can arise naturally and as a by-product of industrial and mining activity, leading to environmental contamination of soil and water (31). Glutaredoxin can transfer electrons from arsenate for energy production while simultaneously detoxifying arsenate in the environment. The presence of diverse mechanisms for resource aquisition and energy generation in *T. alkaliphila* likely confers increased metabolic flexibility, providing a broader range of potential niches and competitive advantages in diverse environments. *T. alkaliphila*’s ability to participate in biogeochemical cycles and detoxify contaminated sites highlights the potential for *T. alkaliphila* to be highly involved in ecological health, processes that should be investigated further for environmental and industrial benefits.

### Draft pangenome calculation

To gather a more holistic view of the capabilities of *T. alkaliphila*, a draft pangenome was calculated. The draft pangenome consists of 4,285 genes, 35.3% of which accounted for the *T. alkaliphila* core genome (i.e., genes present in all 100% of genomes) while shell genes (15% to 95% of genomes) and cloud genes (less than 15% of genomes) accounted for 26.1% and 38.6%, respectively (Fig. 4B and C; Table S5). Outside of core genes involved with central metabolism discussed previously and essential genetic information processing (e.g., DNA replication), *T. alkaliphila* strains carry other functional capacities that may shed light on their lifestyle, community interactions, and ecological roles. For example, all strains carry a copy of two different autoinducer-2 (AI-2) family transporters. These transporters have been identified in other species to function as exporters for the AI-2 quorum sensing molecule used for cell-to-cell communication and as a Na^+^(Li^+^)/H^+^ antiporter for environmental survival (32), specifically high saline-alkaline stress in which *T. alkaliphila* can thrive. *T. alkaliphila* has not been shown to participate in this type of quorum sensing as it does not contain the genetic machinery to create AI-2 molecules or any regulatory proteins that have been shown to be controlled by AI-2. Interestingly, a LuxR transcriptional regulator that is acted on by a different type of quorum sensing molecule, acyl-homoserine lactone (AHL), was detected in just two strains: B4199 and DF92-3, but the ability to produce AHL was not detected. Nevertheless, the detection of LuxR suggests that some strains of *T. alkaliphila* may have the capability to detect and respond to signaling molecules produced by other organisms. *T. alkaliphila* strains also contain many transporters, transporter-binding, and storage proteins for nutrient acquisition in competitive environments, such as for polyamines, essential metals, heme, siderophores, and amino acids. Some of the proteins that were only detected in a few genomes included chemotaxis protein CheW with no other chemotaxis machinery, type IV secretion systems with bacterial conjugation proteins used to transfer DNA, and type V secretion systems that contribute to colonization and virulence. Many strains also contain different insertion sequence transposases, suggesting that *T. alkaliphila* genomes have plasticity and may shuffle their genomes under stress to acquire new adaptions and exchange genes with their community to increase genetic variation. While every genome except strain C6819 had some form of transposase, the strains isolated from chickens contained the majority, suggesting *T. alkaliphila* genome plasticity may be correlated to their habitat. One hypothesis is that due the agricultural setting, chicken isolates were more frequently passaged through environmental sources and hosts leading to increased pressure for adaptation and opportunity for interaction with diverse organisms in which to exchange genes. However, it is important to note, that a draft pangenome calculated from 10 genomes with an average completion of 83.7% likely skews the calculation of the core, shell, and cloud genes. The chicken isolate genome assemblies contain more cloud genes but are more fragmented than the human genome assemblies. Genome fragmentation can result in spuriously identified genes that would normally not be included or would be collapsed together in a more continuous assembly. Because of this, the pangenome calculated here should be considered a draft that should be improved upon as more data on *T. alkaliphila* is collected.

### Virulence factor, bacterial toxin, other secondary metabolite prediction

To determine the potential pathogenicity of *T. alkaliphila*, predicted virulence factors and bacterial toxins were identified from open reading frames (ORFs) within strain genomes (Tables 2 and 3; Tables S6 to S8). These virulence factors and toxins were further screened for the presence of signal peptides used for targeting proteins (henceforth referred to as signaled proteins) for translocation to the membrane for secretion or insertion (33). It was found that distributions of virulence factors and toxins were relatively even across all strains. On average, *T. alkaliphila* strains contained 148.3 +/− 2.07 standard error (SE) signaled virulence factors and 387.6 +/− 4.33 SE non-signaled virulence factors, and 13.0 +/− 0.62 SE signaled toxins and 26.2 +/− 1.08 SE non-signaled toxins. Across all genomes, 167 ORFs were predicted to be both a toxin and virulence factor with 71 of those signaled for translocation and 96 not signaled (Table 2). These toxins and virulence factors were made up of 17 separately annotated proteins (Table 3; Table S8). The outer membrane protein TolC is responsible for exporting small molecules (34), including toxins and antibiotics, and was the most abundant toxin-virulence factor detected in *T. alkaliphila* strains. The *tolC* gene was found in all genomes and in multiple copies which is common with *Pseudomonas* species within the same family as *T. alkaliphila*. Most *tolC*-assigned ORFs also included a signal peptide which would aid in translocation of the TolC protein to the appropriate location in the outer membrane. TolC has been implicated in the export of multiple toxins detected in *T. alkaliphila* strains, including structural toxin protein RtxA1, hemolysin III, hemolysin A, and putative hemolysin, and associated with AcrB/AcrD/AcrF family multi-drug efflux pumps (35, 36). RtxA1 and hemolysins are essential for pathogens and opportunistic pathogens for tissue invasion and lysis of red blood cells (i.e., hemolysis) for nutrient acquisition (37). Additionally, phospholipase D (PLDc_2) was detected in all strains. Phospholipase D in prokaryotes has been associated with DNA hydrolysis and lipase activity, and as a toxin (38). In *Pseudomonas*, it is not clear whether phospholipase D has a role in pathogenesis or cellular homeostasis. Interestingly, some toxins and virulence factors were not evenly detected in human and chicken hosts. For example, the Zot enterotoxin responsible for increasing intestinal permeability in mammals was only found in chicken isolates (39), while insulinase, which has been potentially attributed to host cell invasion, was only detected in human isolates (40). It is unclear why *T. alkaliphila* encodes and maintains various toxins and virulence factors since no disease has been attributed to *T. alkaliphila*; however, one hypothesis is that these factors are primarily activated in competitive environmental instances such as decomposition after host death rather than pathogenesis. Further investigation into what conditions *T. alkaliphila* produces transcripts or toxin and virulence factor proteins is warranted to elucidate this activation.

**TABLE 2:** Virulence factor and toxin prediction summary from PathoFact^*a*^

	Signaled toxin	Non-signaled toxin	Non-toxic
Signaled virulence factor	71	0	1,412
Non-signaled virulence factor	0	96	3,780
Potential signaled virulence factor	22	0	1,578
Potential non-signaled virulence factor	0	116	8,647
Non-virulent	0	47	7,000

^*a*^ The presence of toxin domains, virulence factors, and signal peptides is predicted to classify genomic products. The product is considered non-toxic if a toxin domain is not predicted to be present, non-virulent if virulence factors are not predicted to be present, and non-signaled if a signal peptide sequence is not present.

**TABLE 3:** Proteins identified by PathoFact as both virulence factors and bacterial toxins^*a*^

Protein	Total count	No. genomes present(human/chicken)	Signal peptide presence	Description
ACR_tran	10	10 (7/3)	No	AcrB/AcrD/AcrF family
APH	5	5 (3/2)	No	Phosphotransferase enzyme family
DSPc	6	6 (6/0)	Yes	Dual specificity phosphatase, catalytic domain
Epimerase_Csub	2	2 (1/1)	No	UDP-glucose 4-epimerase C-term subunit
Exo_endo_phos	18	10 (7/3)	No	Endonuclease/exonuclease/phosphatase family
HcnC	16	9 (6/3)	No	Hydrogen cyanide synthase
HlyA	1	1 (0/1)	No	Hemolysin A
HlyIII	7	7 (6/1)	Yes	Hemolysin III
PapC_C	1	1 (0/1)	Yes	Papc C-terminal domain
Peptidase_M16	3	3 (3/0)	Yes	Insulinase (peptidase family M16)
Peptidase_M16_C	3	3 (2/1)	Yes	Peptidase M16 inactive domain
PITRM1, PreP, CYM1	3	3 (1/2)	Yes	Presequence protease
PLDc_2	20	10 (7/3)	Yes (10)/ No (10)	PLD-like domain
RtxA1	10	10 (7/3)	No	Structural toxin protein (hemagglutinin\/hemolysin)
TlyC	11	10 (7/3)	No	Putative hemolysin
TolC	49	10 (7/3)	Yes (38)/ No (11)	Outer membrane protein
Zot	2	2 (0/2)	No	Zona occludens toxin

^*a*^ The total count of hits, the number of genomes the protein was found, whether a signal peptide was predicted and if not unanimous, the number of hits a signal peptide was found within, and the protein description is provided.

Genome-wide identification and annotation of secondary metabolite biosynthesis gene clusters in *T. alkaliphila* strains revealed eight secondary metabolites (Table S9). Four secondary metabolites were detected in all strains: Pf-5 pyoverdine, lankacidin C, lagriene, and an unidentified ribosomally synthesized and post-translationally modified peptide (i.e., RiPP) which can have broad biological function. Pf-5 pyoverdine is an iron siderophore characterized from *Pseudomonas protegens* Pf-5 (formerly known as *Pseudomonas fluorescens* Pf-5), and other similar pyoverdines have been characterized from various *Pseudomonas* species including *Pseudomonas aeruginosa* (41, 42). Iron is an essential element to all life needed for growth and development and serves as a co-factor to many enzymes. Siderophores, such as pyoverdines, are specialized molecules produced by bacteria to bind, acquire, and transport ferric iron from their environment. The ability to produce and take up siderophores serves as a competitive advantage to organisms in iron-limiting ecosystems such as soil and hosts. Pyoverdine has several known functions that contribute to virulence, biofilm formation, and the ability to outcompete soil-borne pathogens (41–44). Lankacidin C and lagriene are known antimicrobial agents produced by the soil-dwelling bacteria *Streptomyces* and *Burkholderia gladioli* bacteria associated with plants, insects, and soil, respectively (45, 46). Plipastatin, a known antimicrobial lipopeptide produced by *Bacillus*, was predicted as a secondary metabolite produced by 90% (9/10) of strains. Plipastatin was not detected in strain DF46-2-2; however, a similar lipopeptide, fengycin, was solely detected in this strain, demonstrating that all strains carry a variation of antimicrobial lipopeptides with broad antagonistic effects on soil-borne fungi (47). Additionally, two antimicrobials, plipastatin and an unidentified β-lactone, were detected in 90% (9/10) of the strains (individually absent from DF46-2-2 and DF95-5, respectively). Lastly, an unidentified β-lactone antimicrobial was detected in nine strains, while an aryl polyene pigment related to a natural product of *Aliivibrio fischeri* responsible for protection against reactive oxygen species and biofilm formation was detected in three strains (48). Taken together, these results suggest that *T. alkaliphila* comes armed for microbial warfare via indirect (e.g., siderophores) and direct (e.g., toxins, antimicrobials) competitive mechanisms. These functions likely also aid in increasing the environmental range of *T. alkaliphila* to include animal hosts and may be advantageous to the decomposition of the same host, an environment from which *T. alkaliphila* has been detected (12, 13).

### Antibiotic resistance-associated gene identification

Exploration of the antimicrobial defensive mechanisms found in *T. alkaliphila* identified 14 genes associated with antibiotic resistance across all strains (Table S10). Out of the 14 genes, three were detected in every strain as core antibiotic resistance genes: *adeF*, *rsmA*, and *qacJ*. The gene *adeF* encodes AdeF, the membrane fusion protein of the multidrug efflux complex AdeFGH. This complex has been demonstrated to provide resistance to broad-spectrum antibiotics tetracycline and fluoroquinolones (49). Tetracycline-specific resistance genes *tet59* and *tetX6* were also detected in three and two strains, respectively. Unsurprisingly, these were the strains isolated from chicken intestines due to their ability to grow in the presence of tetracycline (4). Although *tet59* and *tetX6* were not universally detected, the presence of *adeF* suggests that tetracycline resistance may be a common trait of *T. alkaliphila*. Tetracycline is naturally produced by *Streptomyces*—a soil bacterium associated with the decomposition of plant matter. The universal presence of tetracycline resistance in *T. alkaliphila* suggests that *T. alkaliphila* may have adapted to better compete against *Streptomyces* in decomposition environments. The *Pseudomonas aeruginosa* post-transcriptional regulator RsmA encoded by *rsmA* is a global regulator known to mediate virulence factors and pathogenicity determinants such as biofilm formation, type III secretion systems (T3SS), motility, and antibiotic resistance (50). RsmA positively regulates T3SS and negatively affects the expression of multidrug efflux pumps for fluoroquinolone, diaminopyrimidine, and phenicol antibiotics, thus acting as a cellular balance for virulence and antibiotic resistance. The final gene associated with antibiotic resistance found in all strains, *qacJ*, is responsible for a small antibiotic efflux pump that provides resistance to quaternary ammonium compounds (QACs). QACs are found in clinical, commercial, and household disinfecting products and antiseptics. The other antibiotic resistance genes detected across strains, but not universally, included genes associated with aminoglycoside, sulfonamide, nucleoside, and macrolide resistance.

### Detection of viruses, Clustered Regularly Interspaced Short Palindromic Repeats (CRISPR) loci, and mobile genetic elements

Prediction of prophage sequences within *T. alkaliphila* genomes found six total viruses (Table S11). Five prophage assemblies were of medium and high quality while one assembly was considered poor quality and subsequently removed from the analysis. High-quality prophage genome assemblies had an average viral prediction confidence score of 0.938 +/− 0.01,while the medium-quality assembly had a viral score of 0.896. Four of the medium- and high-quality prophage assemblies were proviruses under the Caudoviricetes family and one provirus was under the Inoviridae family. Caudoviricetes had, on average, 43.33 +/− 9.82 genes and viral prediction confidence score of 92.3 +\− 0.01%. A single Caudoviricetes prophage was predicted to be in the B4199 type strain, C6819, E5571, and DF46-2-2. Therefore, Caudoviricetes were detected in isolates derived not only from human hosts but a chicken host as well. Chicken-derived strain DF46-2-2 was the only strain to contain a prophage in the Inoviridae family which contained 13 genes and a viral prediction score of 95.85%. Notably, predicted plasmid genes were only detected in the chicken-derived isolates (Table S12). Twenty-four plasmid fragments were detected with high confidence (91.6 +/− 0.02%); however, 22 of these plasmids did not contain terminal repeats which are necessary for replication and inheritance. Five plasmids are predicted to host antibiotic resistance genes harboring resistance to tetracyclines, aminoglycosides, and macrolides. Conjugative plasmid genes were detected in all chicken-derived strains by the presence of a predicted functional type IV bacterial secretion system that can transport DNA, including plasmids, across bacterial membranes (Table S12). These data suggest that *T. alkaliphila* can participate in horizontal gene transfer leading to increased genomic capability such as antibiotic resistance.

The CRISPR-Cas (CRISPR-associated proteins) system is an acquired immunity system found in bacteria and archaea that protects against viruses and foreign genetic sequences such as plasmids. A CRISPR region consists of short sequences called repeats separated by unique sequences of a similar length called spacers. Spacers are small fragments of viruses, plasmids, and mobile genetic elements used to combat future infection or genetic manipulation. Here, across all *T. alkaliphila* genomes, three class 1 CRISPR-Cas systems were detected (Table S13). Strain C6819 contained a CAS-typeI-F array with 49 spacers with an average spacer size of 32.02 bp. Strain E1086 contained a CAS-typeI-E array with 31 spacers with an average spacer size of 33.03 bp. Strain DF95-5 contained a CAS-typeI-F array with 59 spacers with an average spacer size of 32 bp. Class 1 CRISPR-Cas systems comprise approximately 90% of CRISPR-Cas systems in bacteria and archaea (51). These systems harbor an RNA-guided multiprotein complex with diverse enzymatic activities, including fractionation of foreign nucleic acids and synthesis of messenger molecules. The presence of CRISPR-Cas systems was not correlated with isolation source as both chicken and human strains were represented, although the human isolates were isolated from leg tissue. It is possible that different lineages of *T. alkaliphila* are devoid of CRISPR-Cas systems due to lack of evolutionary need via minimal natural viral predation. In fact, CRISPR-Cas system presence is highly variable across the bacterial kingdom and only approximately 45% of Gammaproteobacterial lineages contain CRISPR-Cas systems (52). Of those present, many of them consisted of subtypes I-E and I-F, as detected here.

### Conclusion

The genomic characteristics of *Thiopseudomonas alkaliphila* shed light on this relatively unknown organism’s metabolic versatility and potential to survive in diverse habitats. *T. alkaliphila* exhibits habitat-specific genomic variations, with strains isolated from chicken intestines differing phylogenetically from those derived from human sources. Some of these differences can be traced back to prophages and transposable elements that may increase genomic plasticity of chicken isolates. Strains derived from humans were the only strains with the genomic potential to convert tetrathionate to thiosulfate which has been shown to be beneficial for organisms in the gut during inflammation; however, it is not clear whether this is the case for *T. alkaliphila*. Furthermore, it cannot be ruled out that phylogenetic and functional pathway differences between the human and chicken isolates are impacted by the more fragmented genome assembly of the chicken isolates. The presence of various complete and nearly complete metabolic pathways, including glycolysis, the Entner-Doudoroff pathway, sulfur oxidation, arsenate reduction, and acetate assimilation, highlights *T. alkaliphila* metabolic versatility and adaptability to different energy sources which may be useful for industrial uses and bioremediation when fully understood. *T. alkaliphila* genomes feature a range of toxins, virulence factors, and secondary metabolites, including antimicrobial agents and siderophores, suggesting its involvement in microbial competition and resource acquisition in competitive environments and potential as an opportunistic pathogen. Lastly, transposases, prophages, plasmids, and CRISPR-Cas systems were identified in *T. alkaliphila* genomes, indicating potential mechanisms for horizontal gene transfer and defense against viral predation. However, since most genomes did not contain CRISPR-Cas systems, it may suggest that (i) alternative defense strategies are utilized, (ii) evolutionary pressures led to the loss of the CRISPR-Cas system, or (iii) viral predation on this species is uncommon. Further investigation, including environmental bacteriophage profiling and isolation, between *T. alkaliphila* and bacteriophages is necessary to determine the range of interactions. This genomic analysis provides a valuable resource for the scientific community into understanding the health and ecological significances and industrial potential of *T. alkaliphila*, emphasizing the need for further exploration of its diverse capabilities. However, the results presented here should be interpreted with caution as these are genomic annotations and predictions that need to be tested and validated in a laboratory setting if the environmental roles and potential pathogenicity of *T. alkaliphila* are to be truly understood. Beneficial first steps of laboratory validation would include isolation of additional *T. alkaliphila* strains from diverse environments, bioreactor experiments to elucidate the role of *T. alkaliphila* in the breakdown of organic matter, and animal model experiments to determine if the infectious dose of *T. alkaliphila* is comparable with closely related opportunistic pathogens.

## MATERIAL AND METHODS

### Data collection

*Thiopseudomonas alkaliphila* (NCBI:txid 1697053), previously *Oblitimonas alkaliphila*, genomes were searched for in the NCBI Assembly database (https://www.ncbi.nlm.nih.gov/assembly) in July of 2023. Twelve genomes were publicly available; however, two genomes were excluded from the NCBI RefSeq collection due to missing tRNA genes (genome assembly ASM126723v1), or the genome was too small and fragmented (genome assembly ASM236031v1). Therefore, comparative genome and pangenome analyses in this study were only performed on the genomes included in the NCBI RefSeq collection (*N* = 10). These isolates included previously unidentified strains from a historical collection (1969–1979) held in the U.S. Special Bacteriology Reference Laboratory at the Centers for Disease Control and Prevention (1, 2). For this study, genomic data were retrieved from NCBI RefSeq as genomic nucleotide FASTA files (downloaded 2023–07-07), genomic amino acid protein FASTA files (downloaded 2023–07-07), and GenBank version 3 (GFF3) files (downloaded 2023–07-10).

### Average nucleotide identity and GBDP phylogenetic tree

ANI with pairwise comparison between all NCBI RefSeq genomic nucleotide data was calculated using FastANI v.1.33 via the Genome Taxonomy Database (GTDB; https://gtdb.ecogenomic.org/tools/fastani) with default parameters (15). The NCBI RefSeq genomic nucleotide data were uploaded to the Type (Strain) Genome Server v.391 (https://tygs.dsmz.de) for a whole genome-based taxonomic analysis (53, 54). Phylogenetic trees of *T. alkaliphila* strains with closely related type strains and a strains-only tree were generated. Determination of closest type strain genomes was done in two complementary ways: first, all *T. alkaliphila* strain genomes were compared against all 20,806 type strain genomes available in the TYGS database via the MASH algorithm and the 10 type strains with the smallest MASH distances chosen per *T. alkaliphila* strain genome (55). Second, an additional set of 10 closely related type strains was determined via the 16S rRNA gene sequences. These were extracted from the *T. alkaliphila* strain genomes using RNAmmer and each sequence was subsequently BLAST aligned against the 16S rRNA gene sequences in the TYGS database (56, 57). This was used as a proxy to find the best 50 matching type strains for each *T. alkaliphila* strain genome and to calculate precise distances using the GBDP approach under the algorithm “coverage” and distance formula d_5_ (58). These distances were finally used to determine the 10 closest type strain genomes for each of the *T. alkaliphila* strain genomes. For the phylogenomic inference, all pairwise comparisons among the set of genomes were conducted using GBDP and accurate intergenomic distances were inferred under the algorithm “trimming” and distance formula d_5_ (100 distance replicates calculated for each). An additional GBDP phylogenomic analysis was inferred using the amino acid sequences of the entire proteome. Digital DDH values and confidence intervals were calculated using the recommended settings of the Genome-to-Genome Distance Calculator (GGDC) v.4.0 (54, 58). The resulting intergenomic distances were used to infer a balanced minimum evolution midpoint-rooted tree with branch support via FASTME v.2.1.6.1 including Subtree Pruning and Regrafting (SPR) postprocessing, and branch support was inferred from 100 pseudo-bootstrap replicates each (59). The type-based species clustering using a 70% dDDH radius around each of the type strains and subspecies clustering were done using a 79% dDDH threshold as previously introduced (60). The intergenomic distance matrices are used to calculate the δ statistic value, allowing for assessing the impact of individual genomes on overall treelikeness (53). Genomes with high δ values render the data less treelike, which can correspond to sequence incompleteness/contamination or be related to long-branch attraction. Thus, δ values provide evidence suggesting how specific genome sequences impact the reliability of the phylogenetic tree outcome.

### Metabolic annotation and pangenome calculation

Default parameters for computational tools were used unless stated otherwise. Functional annotation was performed using Prokka (Galaxy version 1.14.6 + galaxy1) using NCBI RefSeq genomic nucleotide FASTA files and adding the “--proteins” flag to provide NCBI RefSeq genomic amino acid protein FASTA files (61, 62). DRAM v.1.4.6 (Distilled and Refined Annotation of Metabolism) was used to profile genomes for metabolisms known to impact ecosystem function across biomes using NCBI RefSeq genomic nucleotide FASTA files (63). Roary (Galaxy version 3.13.0 + galaxy2) was used to calculate the *Thiopseudomonas alkaliphila* pangenome using the NCBI GFF3 files (64). Roary calculated pangenome core genes (present in 99%–100% of strains), soft core genes (95%–99%), shell genes (15%–95%), and cloud genes (0%–15%). Next, Roary provided presence/absence data of each gene with each strain. Roary output was visualized with an adapted version of the base roary_plots.ipynb jupyter notebook located on the Roary GitHub repository (https://github.com/sanger-pathogens/Roary/tree/master/contrib/roary_plots) which can be found on the author’s GitHub repository (https://github.com/BurchamLab/TPA_pangenome) using the TYGS GBDP whole-genome tree containing only the *T. alkaliphila* strains.

### Antibiotic resistance and secondary metabolite annotation

The online Resistance Gene Identifier in conjunction with the Comprehensive Antibiotic Resistance Database (https://card.mcmaster.ca/home) was used to predict resistomes from the NCBI RefSeq genomic nucleotide data based on homology and single nucleotide polymorphism (SNP) models with selection criteria of “Perfect and Strict hits only” and excluding prediction of partial genes (65). Genome-wide identification, annotation, and analysis of secondary metabolite biosynthesis gene clusters in each genome was performed using antiSMASH v.7.0 (https://antismash.secondarymetabolites.org/) from the NCBI RefSeq genomic nucleotide data (66). A “relaxed” detection strictness was selected to detect both well-defined clusters containing all required parts and partial clusters missing one or more functional parts. Virulence factors and bacterial toxins present within genomes were predicted with PathoFact v.1.0 using default parameters (67). Toxin classification level is given depending on toxin and signal peptide predictions; a classification level of 1:signaled toxin when a sequence is predicted to contain a toxin domain as well as contain a signal peptide, and a level of 2:non-signaled toxin when only predicted to contain a toxin domain and not signaled. The product is considered non-toxic if a toxin domain is not predicted. Virulence classification level is given depending on the consensus prediction of the hidden Markov models (HMM) and random forest classifier models along with the prediction for a signal peptide. Virulence classifications are as follows: 1:signaled virulence factor, 2:non-signaled virulence factor, 3:potential signaled virulence factor, 4:potential non-signaled virulence factor, and non-virulent if neither model predicts virulence.

### Bacteriophage, mobile genetic element, and CRISPR loci detection

The National Microbiome Data Collaborative EDGE platform (https://nmdc-edge.org/home) was used to identify viruses and mobile genetic elements using geNomad (68) and extracted metagenome-assembled viral genomes were quality checked using CheckV (69) using default settings. The geNomad classification model assigns a confidence score between 0 (lowest confidence) and 1 (highest confidence) to each prediction. Only confidence scores above 0.7 were reported. If plasmids were found, mobility and conjugative elements were detected in plasmid amino acid sequences using systems modeling and similarity search by MacSyFinder2-based CONJscan 2.0.1 on Galaxy (Galaxy version 3.13.0 + galaxy2) as circular, ordered replicons (70). Antimicrobial genes found within plasmid sequences were annotated with AMRFinderPlus (https://www.ncbi.nlm.nih.gov/pathogens/hmm/) by geNomad. CRISPRCasFinder (https://crisprcas.i2bc.paris-saclay.fr/) was used to detect CRISPR and cas genes from the NCBI RefSeq genomic nucleotide data (71). Acr (anti-CRISPR) proteins encoded from *acr* genes are made by phages and other mobile genetic elements. Acr proteins form operons with transcription regulator genes that encode Aca (Acr-associated) proteins to inhibit the CRISPR-Cas systems of their bacterial hosts. AcrFinder (https://bcb.unl.edu/AcrFinder/index.php) was used to identify known Acr or Aca proteins within the bacterial genomes from the NCBI RefSeq genomic nucleotide and amino acid protein data with the GFF3 file (72).

## SUPPLEMENTAL MATERIAL

Supplemental material spectrum.04157-23-s0001.xlsx Tables S1 to S13.

ASM does not own the copyrights to Supplemental Material that may be linked to, or accessed through, an article. The authors have granted ASM a non-exclusive, world-wide license to publish the Supplemental Material files. Please contact the corresponding author directly for reuse.
